# Complete genome sequence of *Lactococcus garvieae* isolated from a greater amberjack (*Seriola dumerili*) farmed in Japan in 2022

**DOI:** 10.1128/mra.00436-24

**Published:** 2024-07-16

**Authors:** Maiga Salif, Riku Ogawa, Aguri Mikami, Masanori Daibata, Masayuki Imajoh

**Affiliations:** 1 Department of Bioresource Production Science, The United Graduate School of Agricultural Sciences, Ehime University, Ehime, Japan; 2 Faculty of Agriculture and Marine Science, Graduate School of Integrated Arts and Science, Kochi University, Kochi, Japan; 3 Department of Microbiology and Infection, Kochi Medical School, Kochi University, Kochi, Japan; California State University San Marcos, San Marcos, California, USA

**Keywords:** *Lactococcus garvieae*, greater amberjack

## Abstract

The complete genome sequence of *Lactococcus garvieae* KN22525, isolated in May 2022 from a diseased greater amberjack (*Seriola dumerili*) with a gross indication of body surface redness in Nomi Bay, Japan, was determined. Multilocus sequencing typing revealed that KN22525’s genotype was sequence type ST95.

## ANNOUNCEMENT

*Lactococcus garvieae* infection has resulted in sustained economic losses in *Seriola* species aquaculture in Japan since 1974 (1). According to the Ministry of Agriculture, Forestry, and Fisheries of Japan, the estimated economic damage between 2017 and 2021 was 1,180–1,973 million yen (2). Among *Seriola* species, greater amberjack (*Seriola dumerili*) is prominent, with Japan being the global leader in production since the initiation of farming in the 1970s, primarily relying on imported juveniles from China (3, 4). Nomi Bay (33°22'12.2"N, 133°18'22.8"E), characterized by its ria coast facing the Pacific Ocean, is located in the south of Shikoku Island, Japan (5). A diseased dead greater amberjack with a gross indication of body surface redness was provided for diagnosis by a fish farmer in Nomi Bay in May 2022, less than a month after its import from China. *Lactococcus garvieae* was isolated from the brain via brain–heart infusion broth (BHI, BD Difco, USA) containing 1.5% NaCl (BHI salt) agar plates by incubation at 25°C overnight. Herein, we report the complete genome sequence of the isolate KN22525.

Frozen stocks of KN22525 were cultured on BHI salt agar plates at 25°C overnight. A single colony was selected and incubated with shaking in 10 mL of BHI salt broth at 25°C overnight. Following high-speed centrifugation, genomic DNA was extracted from the bacterial pellets using the Wizard HMW DNA Extraction Kit (Promega, USA) and used for Illumina and Nanopore sequencing. For Illumina sequencing, genomic DNA was fragmented and processed to achieve an average size of 330 bp using the QIAseq FX DNA Library Kit (Qiagen, USA). After adapter ligation, 20 pM pooled libraries were sequenced on an Illumina MiSeq (MiSeq Reagent Kit v3; 150 cycles, USA), generating 75-bp paired-end reads. MiSeq raw reads with Phred-encoded quality scores above 30 were selected for assembly using Trim Galore v.0.6.5 (6). For Nanopore sequencing, genomic DNA was treated with the 1D Ligation Sequencing Kit (SQK-LSK109, Oxford Nanopore Technologies, USA) with Native Barcoding Expansion (EXP-NBD104) without sheareing and size selection to avoid fragmentation. After adapter ligation, the pooled libraries were sequenced on a MinION with an R9.4.1 flow cell using MinKNOW software v22.05.5. Base-calling was performed using Guppy v.4.2.3 in the accurate mode implemented in MinION software. MinION raw reads below a minimum quality score of 8 were discarded using NanoFilt v.2.8.0 (7). Unicycler v.0.4.8 (8) was used for hybrid assembly of MiSeq and MinION reads, with its default parameters applied. Genome error correction, circularization, and rotation were implemented in the Unicycler pipeline. Gene annotation, taxonomy verification, and genotyping were performed using the DDBJ Fast Annotation and Submission Tool v.1.2.0 (9) and the multilocus sequencing typing (MLST) databases for *L. garvieae* (10).

The genome characteristics of KN22525 are presented in Table 1. MLST results identified KN22525 as sequence type ST95, similar to strain MS210922A, for which the complete genome sequence was reported previously (11). This strain, also recently isolated from a greater amberjack in Japan, akin to KN22525, was proposed as the third new serotype of *L. garvieae* based on serotyping and genotyping (12).

**TABLE 1:** Characteristics of the complete genome sequence of *L. garvieae* KN22525

Number of contigs	1
CheckM completeness/contamination (%)	96.05/1.68
Structure	Circular chromosome
Number of MiSeq reads	2,252,536
Number of MinION reads (N50 value (bp))	16,906 (15,986)
Chromosome size (bp)	1,976,750
GC content (%)	39.0
Coverage (×)	334
N50 value (bp)	1,976,750
Number of coding sequences	1,881
Number of rRNAs	16
Number of tRNAs	58
Whole-genome sequence accession no.	AP031391
DRA accession nos. of Illumina MiSeq and MinION data	DRX528542 and DRX528543
ST type	95

## Data Availability

The complete genome sequence of *L. garvieae* KN22525 is available at the National Center for Biotechnology Information under accession number AP031391. Raw sequence data from Illumina MiSeq and MinION are available at DDBJ Sequence Read Archive under DRA accession numbers DRX528542 and DRX528543.

## AFTER EPUB

*[This article was published on 16 July 2024 with an error in the title. The title was corrected in the current version, posted on 19 July 2024.]*
